# Mullerian cyst of the posterior mediastinum: utilizing indocyanine green for thoracic duct visualization during robotic-assisted resection

**DOI:** 10.1093/icvts/ivaf122

**Published:** 2025-05-27

**Authors:** William A Hardy, Jonathan Waxman

**Affiliations:** Department of Surgery, BayCare Health System, Tampa, FL, USA; Department of Cardiothoracic Surgery, BayCare Health System, Tampa, FL, USA

**Keywords:** posterior mediastinum, mediastinal Mullerian cyst, cyst of Hattori, thoracic duct, indocyanine green

## Abstract

We describe a case of a 45-year-old female who underwent robot-assisted resection for a posterior mediastinal cyst abutting the thoracic duct. During this operation, near-infrared fluorescence lymphography with indocyanine green was successfully utilized for real-time intraoperative visualization of the thoracic duct. This technique should be considered when operating on structures near the thoracic duct to avoid and promptly detect iatrogenic injury.

## INTRODUCTION

Mullerian mediastinal cysts are rare benign lesions characterized by the presence of ectopic Mullerian tissue in the posterior mediastinum [1, 2]. We describe a case of a mediastinal Mullerian cyst abutting the thoracic duct, which was resected using near-infrared indocyanine green (ICG) lymphography of the thoracic duct.

## CASE PRESENTATION

**Figure 1:**
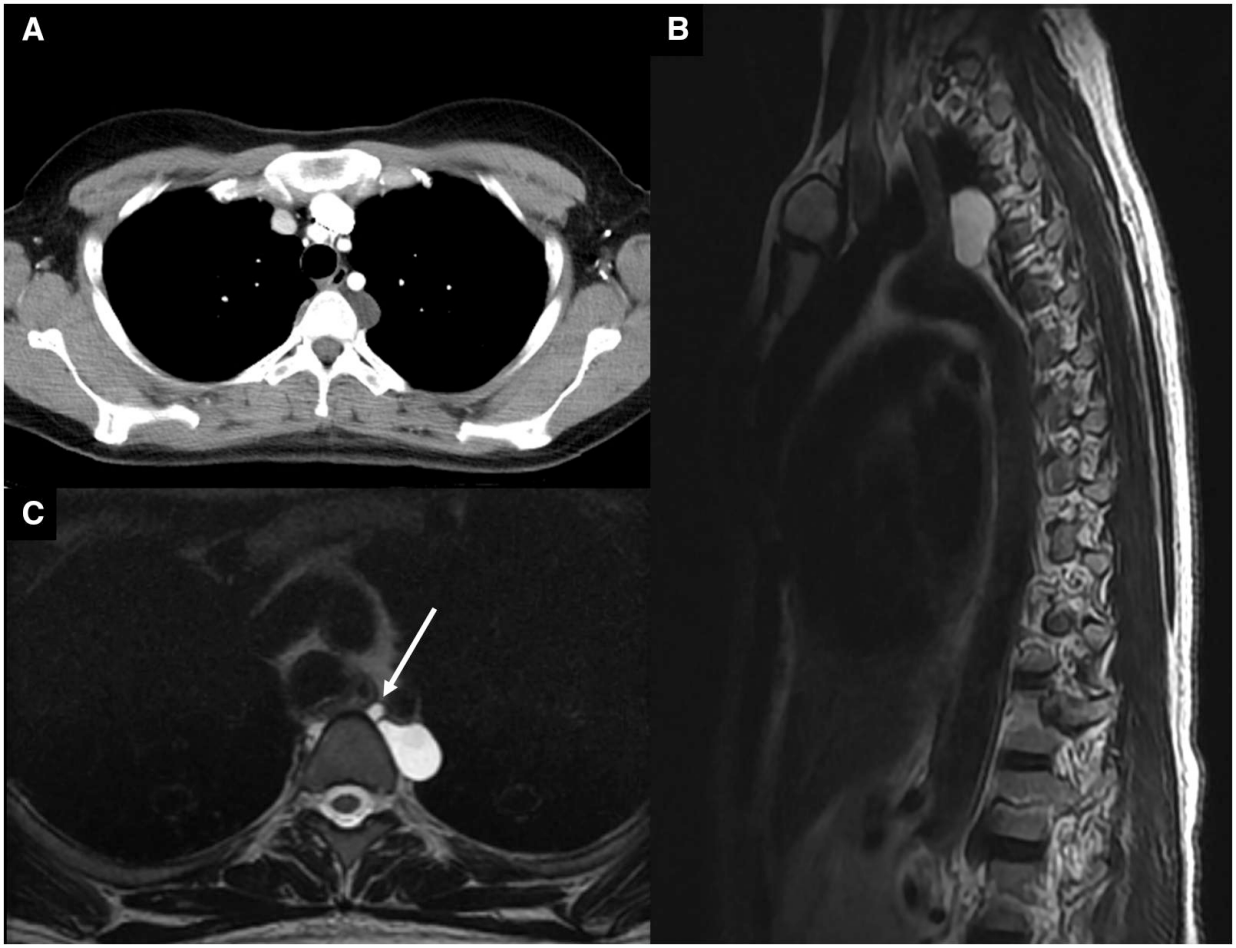
(**a**) Computed tomography of mediastinal Mullerian cyst. (**B**) T2-weighted MRI sagittal view showing a cyst between the left subclavian artery and the descending aorta. (**C**) T2-weighted MRI axial view demonstrating cyst and thoracic duct (arrow)

A 45-year-old female with a history of radiculopathy secondary to L4–L5 disc disease was referred to our clinic for evaluation of a posterior mediastinal cyst found during magnetic resonance imaging (MRI) of the thoracic spine. This MRI demonstrated a 3.0 cm left-sided unilocular cystic lesion in the posterior mediastinum abutting the thoracic duct at the level of T3-T4 (Fig. 1). Computed tomography (CT) similarly demonstrated the same cystic lesion, which was non-enhancing. Her physical exam was unremarkable.

**Figure 2:**
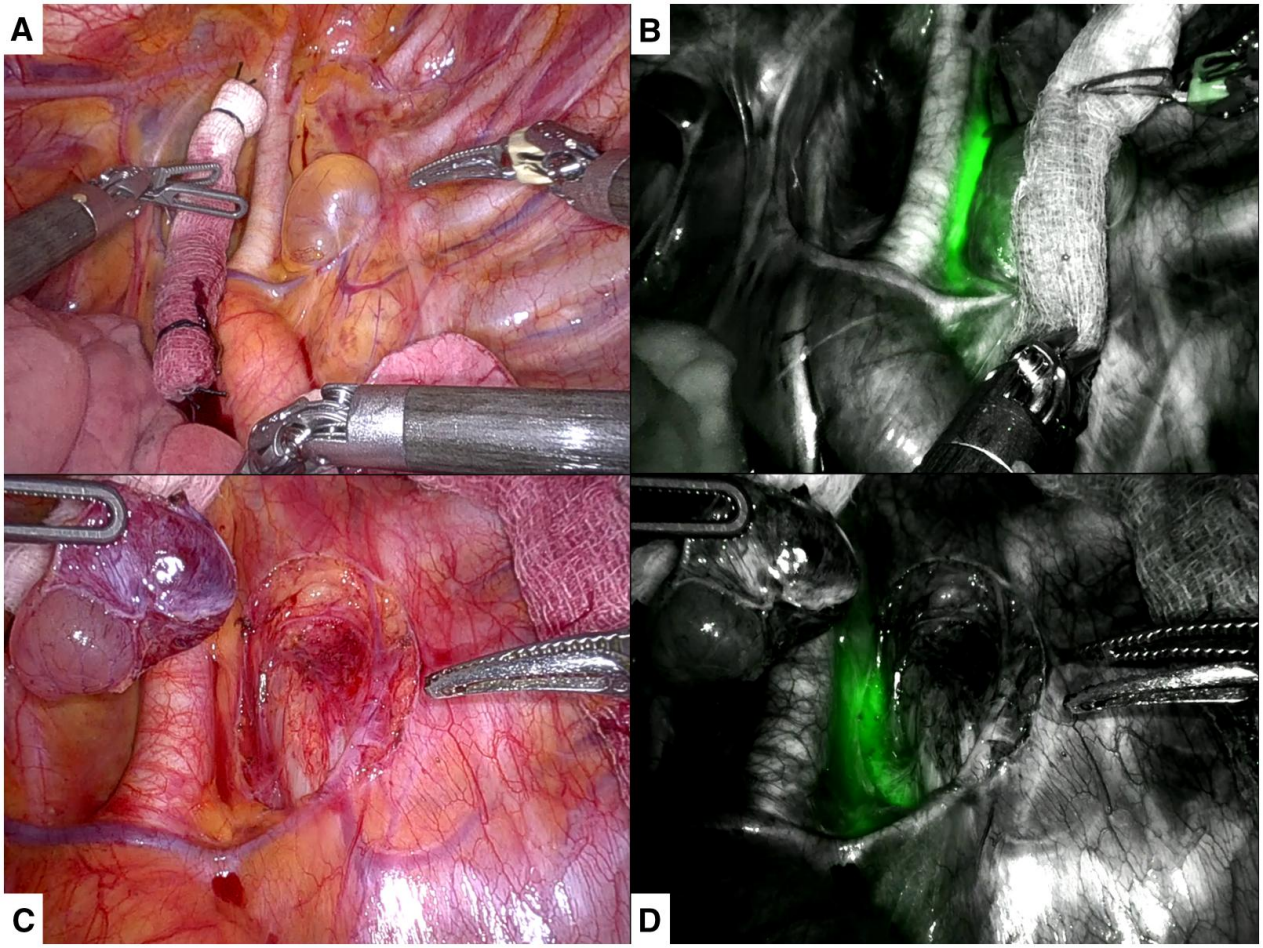
(**a**) Cyst located between the left subclavian artery and descending aorta. (**B**) Near-infrared fluorescence imaging showing the proximity of the thoracic duct to the cyst. (**C and D**) Resection bed without (C) and with (D) near-infrared fluorescence imaging after resection

The decision was made to surgically excise the cyst to definitively establish a diagnosis. Preoperatively, the patient underwent intranodal administration of ICG to facilitate thoracic duct identification. Using real-time ultrasound guidance, a 25-gauge spinal needle was inserted between the cortex and medulla of a right superficial inguinal lymph node and 12.5 mg of ICG in 2.5 ml sterile saline was injected. This procedure was repeated on the left side, for a total administration of 25 mg of ICG. The patient was then brought to the operating room to undergo robotic-assisted thoracoscopic resection. Upon mobilization of the left upper lobe, the cystic mass was identified above the aortic arch behind the left subclavian artery (Fig. 2). Near-infrared fluorescence imaging (Firefly technology) was then used to visualize the thoracic duct and guide the dissection. The mediastinal pleura was opened and the loose attachments of the cyst to the mediastinum were dissected using bipolar cautery. Firefly was again utilized after the cyst was completely resected, and no leakage of ICG from the thoracic duct was observed.

The patient recovered uneventfully and was discharged home on postoperative day 1. An intrapleural drain was left intraoperatively and was removed several hours later once regular diet had been resumed and no chyle leak was identified. At 2-week follow-up, the patient reported no incisional pain. Chest radiography was negative for pneumothorax or pleural effusion. Pathologic examination demonstrated the cyst to be unilocular and lined by benign ciliated columnar epithelium. Immunohistochemical stains were positive for paired box gene 8 (PAX8) and oestrogen receptor (ER), confirming the diagnosis of a Mullerian cyst.

## DISCUSSION

Mullerian mediastinal cysts were first described by Hattori in 2005 [2]. They often occur in women aged 40–60 years, and are located in the posterior mediastinum at the levels of T4-T6 [1]. Radiographically, they appear as unilocular cysts abutting the vertebral body [3]. The lesions appear hypodense on CT and hyperintense on T2-weighted MRI. Ultimately, a definitive diagnosis is made with immunohistochemical staining after surgical resection. Positive staining for ER, progesterone receptor (PR), or PAX8 are specific for Mullerian cysts [1]. In addition to confirming a diagnosis, resection is recommended to obviate the risk of malignant transformation.

Thoracic duct visualization using ICG is a recently developed technique intended to mitigate thoracic duct injuries and has primarily been utilized for oesophagectomy [4]. ICG is especially advantageous because it allows for real-time intraoperative imaging. Additionally, many video-assisted thoracoscopic surgery and robotic platforms are equipped with integrated near-infrared fluorescence imaging systems. Compared to methylene blue, ICG is superior because near-infrared radiation penetrates tissue more deeply than visible light, enabling visualization of the thoracic duct even when it is not completely exposed [5]. Lymphangiography is less practical than ICG because it requires the use of intraoperative fluoroscopy.

In our case, intraoperative visualization of thoracic duct was achieved without difficulty, despite a 2-hour interval between ICG administration and intraoperative visualization. In their study of ICG use for thoracic duct identification during oesophagectomy, Vecchiato *et al.* reported a time interval of 35–80 min between administration and visualization, additionally noting a median operative time of 258 min with ICG fluorescence detectable at the end of all procedures [4]. Together, these findings and our experience suggest that thoracic duct fluorescence sufficiently persists for at least several hours following injection.

The limitations of ICG fluorescence for thoracic duct identification are not well characterized, though they conceivably exist. For instance, if the thoracic duct lies posterior to a deep-seated mediastinal cyst, the tissue penetrance of near-infrared imaging may be exceeded, and the thoracic duct may not be sufficiently identified. Additionally, disruptions in lymphatic drainage could delay or prevent ICG from reaching the thoracic duct, a possibility that should be considered in patients with lymphedema or chylous ascites.

Real-time intraoperative visualization of the thoracic duct with ICG is a recent advancement in thoracic surgery. This report is the first to detail its application during resection of a posterior mediastinal cyst. The technique should be considered when operating near the thoracic duct to help avoid and promptly detect iatrogenic injury.

## Data Availability

The study data will be available upon reasonable request to the corresponding author.
